# Probe-based association analysis identifies several deletions associated with average daily gain in beef cattle

**DOI:** 10.1186/s12864-018-5403-5

**Published:** 2019-01-10

**Authors:** Lingyang Xu, Liu Yang, Lei Wang, Bo Zhu, Yan Chen, Huijiang Gao, Xue Gao, Lupei Zhang, George E. Liu, Junya Li

**Affiliations:** 1 Innovation Team of Cattle Genetic Breeding, Institute of Animal Sciences, Chinese Academy of Agricultural Sciences, Beijing, 100193 China; 2 Farm Animal Genetic Resources Exploration and Innovation Key Laboratory of Sichuan Province, Sichuan Agricultural University, Chengdu, 611130 China; 3 Beijing Genecast Biotechnology Co., Beijing, 100191 China; 4 U.S. Department of Agriculture-Agricultural Research Services, Animal Genomics and Improvement Laboratory, Beltsville, MD 20705 USA

**Keywords:** Copy number variation, Average daily gain, Probe-based association, Positive selection, Beef cattle

## Abstract

**Background:**

Average daily gain (ADG) is an important trait that contributes to the production efficiency and economic benefits in the beef cattle industry. The molecular mechanisms of ADG have not yet been fully explored because most recent association studies for ADG are based on SNPs or haplotypes. We reported a systematic CNV discovery and association analysis for ADG in Chinese Simmental beef cattle.

**Results:**

Our study identified 4912 nonredundant CNVRs with a total length of ~ 248.7 Mb, corresponding to ~ 8.9% of the cattle genome. Using probe-based CNV association, we identified 24 and 12 significant SNP probes within five deletions and two duplications for ADG, respectively. Among them, we found one common deletion with 89 kb imbedded in LHFPL Tetraspan Subfamily Member 6 (*LHFPL6*) at 22.9 Mb on BTA12, which has high frequency (12.9%) dispersing across population. CNV selection test using V_*ST*_ statistic suggested this common deletion may be under positive selection in Chinese Simmental cattle. Moreover, this deletion was not overlapped with any candidate SNP for ADG compared with previous SNPs-based association studies, suggesting its important role for ADG. In addition, we identified one rare deletion near gene Growth Factor Receptor-bound Protein 10 (*GRB10*) at 5.1 Mb on BTA4 for ADG using both probe-based association and region-based approaches.

**Conclusions:**

Our results provided some valuable insights to elucidate the genetic basis of ADG in beef cattle, and these findings offer an alternative perspective to understand the genetic mechanism of complex traits in terms of copy number variations in farm animals.

**Electronic supplementary material:**

The online version of this article (10.1186/s12864-018-5403-5) contains supplementary material, which is available to authorized users.

## Background

Genomic structural variants mainly comprised of copy number variations (CNVs) in the form of large-scale insertions and deletions, as well as inversions and translocations [1]. CNVs involve more genomic sequence as compared to nucleotide polymorphisms (SNPs), thus they have potentially larger effects, including alternating gene regulation and dosage, contributing to gene expression and risk for normal phenotypic variability [2–5].

High-throughput SNP genotyping arrays have been widely used in genome-wide studies. While these arrays have limited capacity to assess the effects of rare single-site variants, they can be readily used to identify large copy number variations, even if they occur in only a few subjects [6]. There are tremendous evidences showing that other genetic variants like copy number variations may affect complex traits, including short stature and anthropometric traits in human [7, 8]. For instance, one recent study suggested that a 45 kb deletion was associated with the body mass index in humans, which also reflects neuronal influence of the deletion on body weight regulation [9]. Previous study identified several genes (e.g., *MC4R*, *FIBIN*, and *FMO5*), harboring both common and rare variants which may affect body size and anthropometric traits using a CNV-association analysis in European adults [8].

Considerable attention has turned towards assessing the association between copy number variations and complex traits in farm animals using high-throughput array. In cattle, several studies have found CNVs are likely to be associated with resistance to gastrointestinal nematodes in Angus [10, 11] and residual feed intake, milk production and fertility traits in Holstein cows [12–14]. Also, a recent study described a 660 kb deletion which has antagonistic effects on fertility and milk production in Nordic Red cattle [15]. Thus, detecting CNVs and identifying their potential associations have gradually become an alternative method to comprehensively elucidate the genetic mechanism of complex traits in farm animals.

Average daily gain (ADG) is generally recognized as an economically important growth trait that contributes to the production benefits in the beef industry. Previous studies have identified many QTL regions associated with ADG in various populations [16–25], these studies had utilized multiple methods including SNP-based GWAS, haplotype-based GWAS and gene-based GWAS to test the association for ADG in various populations. However, the molecular mechanism of ADG have not yet been fully explored, partially because most recent studies of ADG are based on SNPs or haplotype alone, and systematic association study for this complex trait based on CNVs is still missing.

In this study, we presented a comprehensive CNV association analysis for ADG in Chinese Simmental beef cattle. Seven CNVs were identified significantly associated with ADG using probed-base association analysis. Notably, we found one common deletion with 89 kb imbedded in *LHFPL6* with high frequency and one rare deletion overlapped with *GRB10* as potential candidate variants for ADG in Chinese Simmental cattle. Further systematic studies indicated the identified common deletion may contribute additional effect to ADG beyond SNPs.

## Results

### CNV identification

We performed CNV analysis with the Illumina Bovine HD BeadChip in Chinese Simmental beef cattle. A total of 234,973 raw CNV events were generated using PennCNV v1.0.4 [26] based on the UMD3.1 genome assembly. After quality control, 61,710 of them in 1079 individuals that met quality thresholds were kept for subsequent analyses. On average, 57.2 CNV events were obtained for each individual, with average length of 3.6 Mb (Additional file 1). These CNVs were merged into 4912 nonredundant copy number variation regions (CNVRs) with a total length of ~ 248.7 Mb, corresponding to ~ 8.9% of the cattle genome.

### Enrichment analysis using CNV-disrupting genes

We further investigated the gene-disrupting CNVs using the DAVID (The Database for Annotation, Visualization and Integrated Discovery) system to check enrichment for these genes. Duplication and deletion were considered separately in current study. We obtained 1863 and 629 genes overlapped with deletion and duplication regions, respectively (Additional file 2). Using DAVID annotation platform, for deletions we found that a significant over-representation of genes related to antigen processing and presentation of peptide or polysaccharide antigen via MHC class II and MHC class II protein complex, while for duplications we found that several genes were enriched in MHC class I protein complex, antigen processing and presentation of peptide antigen via MHC class I, immune response, antigen processing and presentation of peptide or polysaccharide antigen via MHC class II and MHC class II protein complex (Additional file 3).

### CNVs overlap with QTL associated with ADG trait

We next explored the overlap of QTLs on CNV regions (at least 1 bp overlap between them). We retrieved autosomal QTL regions from QTLdb associated with the trait classes ‘Average daily gain’. We found that 356 deletion and 135 duplication regions overlapped with the merged QTL regions for ADG. Among them, deletion regions occupy ~ 14.13 Mb, while duplication regions occupy ~ 4.08 Mb (Additional file 4). These findings imply these CNVs is likely to be used as new potential candidate markers to refine cattle QTLs after validation.

### ADG associated CNVs

**Fig. 1:**
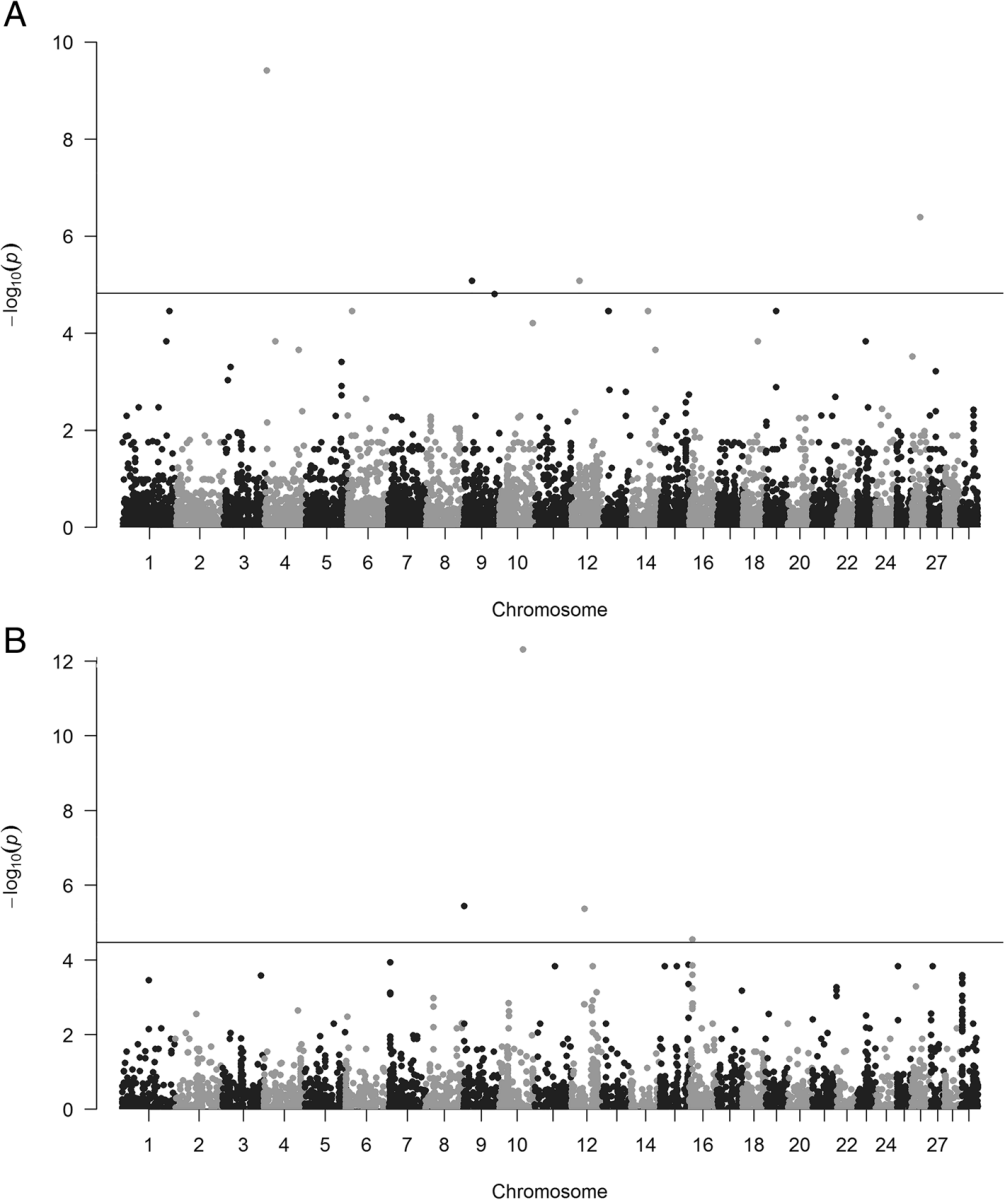
Manhattan plots for probe-based CNV association analysis. **a** Genome wide association results of deletion CNVs for average dairy gain. **b** Genome wide association results of duplication CNVs for average dairy gain. The -log_10_ (*P* value) of each probe (y-axis) in the association-analysis using EMMMAX algorithm is plotted against the genomic position (x-axis)

We carried out probe-based CNV association analysis for ADG, and this approach converts the individual-level CNV calls into population-level probe-based CNV. The probe-based CNV table was generated by running the ParseCNV.pl script with the includePed option implemented in ParseCNV2.0 program [27]. We then conducted an association test for ADG using the mixed linear models plugged in the EMMAX software. In this study, deletion-only and duplication-only models were utilized to separately detect the associated deletion and duplication regions. We obtained 62,952 and 21,802 probes within deletion and duplication regions for subsequent association analysis, respectively. Using mixed linear models, we identified 24 and 12 significant SNP probes within deletion and duplication region based on genome-wide significant thresholds, where *P* values were set to 1.59E-05 for deletions and 4.59E-05 for duplications as suggested by ParseCNV [27] (Additional file 5). Manhattan plots for probe-based CNV association analysis of deletion and duplication CNVs for average dairy gain were presented in Fig. 1a and b.

Based on the significant level of probes in CNVRs, we defined the candidate regions as the CNVRs with at less two significant probes under the suggestive significant level. The 36 significant probes were detected within 5 deletion CNVRs and 2 duplication CNVRs (CNV with only one significat probe was not included), which ranged from 9265 bp to 89,050 bp in length (Table 1). However, we found two top probes associated with ADG using a FDR multiple-correction (*P* < 0.01). One probe with deletion located at 5.1 Mb on BTA4 (P_FDR_ = 3.02E-04), and one probe within duplication located at ~ 68.1 Mb on BTA10 (P_FDR_ = 1.54E-04). Among the identified seven CNVRs, we identified 2 deletions imbedded with genes including *LHFPL6* and *SORCS3*. One deletion within *LHFPL6* on BTA12 shows a highest frequency of 12.9%, while other deletions display relatively low frequencies and located at BTA4, BTA9 and BTA12.

**Table 1:** Candidate copy number variation regions associated with average dairy gain for beef cattle

CNV Type	BTA	Start	End	Length (bp)	Count of significant probes	Distance (bp)	Candidate Genes
Del	4	5,081,669	5,090,934	9265	5	13,965	*GRB10*
Del	9	25,021,405	25,050,866	29,461	10	212,896	*CENPW*
Del	9	90,417,787	90,428,353	10,566	3	161,602	*ESR1*
Del	12	22,890,419	22,979,469	89,050	2	within	*LHFPL6*
Del	26	25,675,473	25,682,667	7194	4	within	*SORCS3*
Dup	9	2,688,360	2,760,007	71,647	8	1,972,937	*PHF3*
Dup	10	68,148,124	68,150,780	2656	4	37,825	*ATG14*

Beside deletions, we also identified two candidate duplications for ADG. However, no gene was found within these duplication regions. In addition, we found one duplication with 125 kb displaying a frequency of 0.74% in our population. One significant probe in duplication located at the upstream of *R3HDM2*, but only one significant probe was detected for this duplication.

**Fig. 2:**
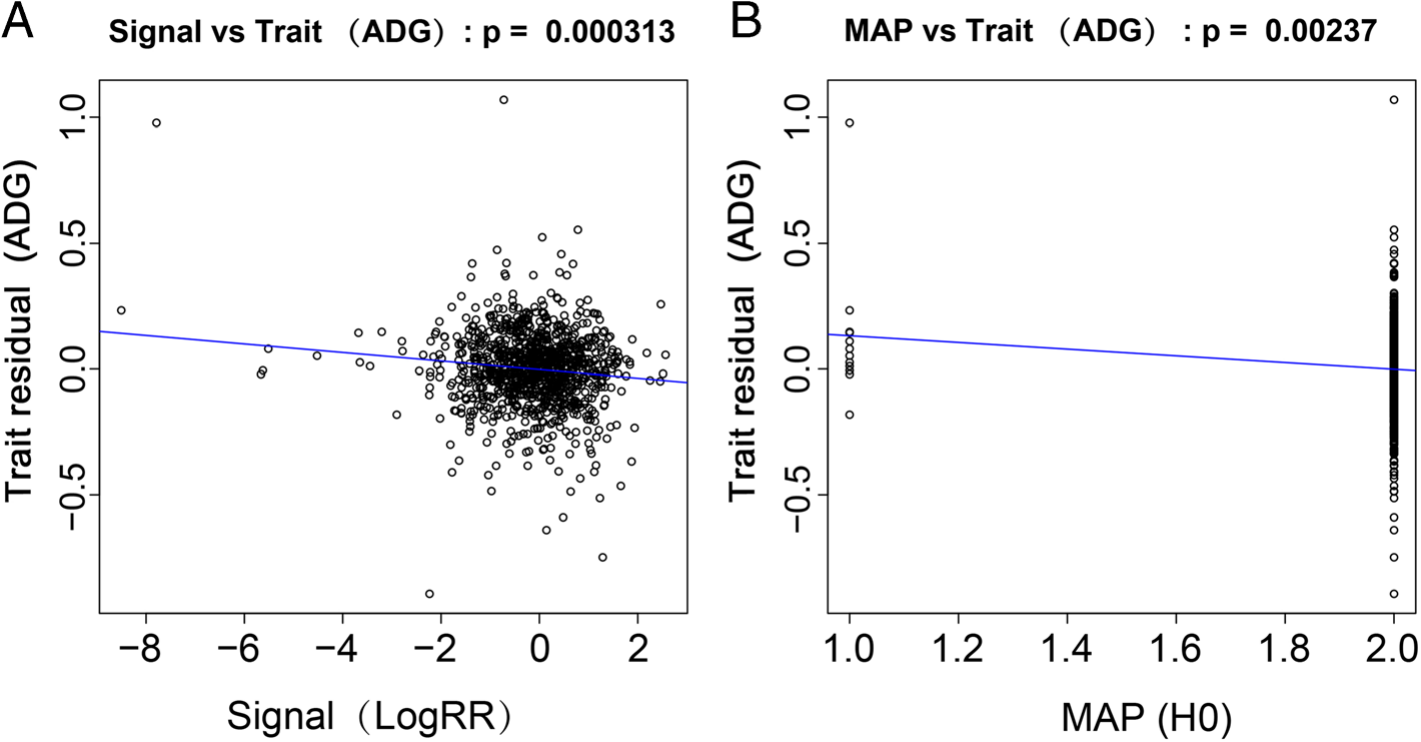
Region association of ADG for CNV region at 5.1 Mb on BTA4. **a** The adjusted trait residuals against signal (LogRR) for CNV region at 5.1 Mb on BTA4. **b** The adjusted trait residuals against copy number state (MAP) estimated by mixture model assignment

Besides the probe-based approach for CNV association, we also examined CNV calls affecting potential regions for ADG using an alternative region-based method, which have been previously described in CNVtools [28]. Using region-based association, we only observed one associated rare deletion with 9265 kb at 5.1 Mb on BTA4, which was located at ~ 14 kb upstream of *GRB10* (Growth Factor Receptor Bound Protein 10) (Fig. 2a and b), while no significant signal was observed among other candidate CNV regions.

In addition, to ensure reliability of our CNV detection method, we randomly selected seven identified CNVs representing different types for quantitative PCR (qPCR), and examine eight samples which contain each of seven CNVs. Two distinct pairs of primers were designed using Primer 3.0 for each detected CNV (Additional file 6). Our analysis showed that the validation rates of the eight samples varied from 71.43 to 100% with an average of 85.71%, which were comparable to our earlier results and other studies [29–33].

### Selection estimation and sequencing validation for one common deletion

**Fig. 3:**
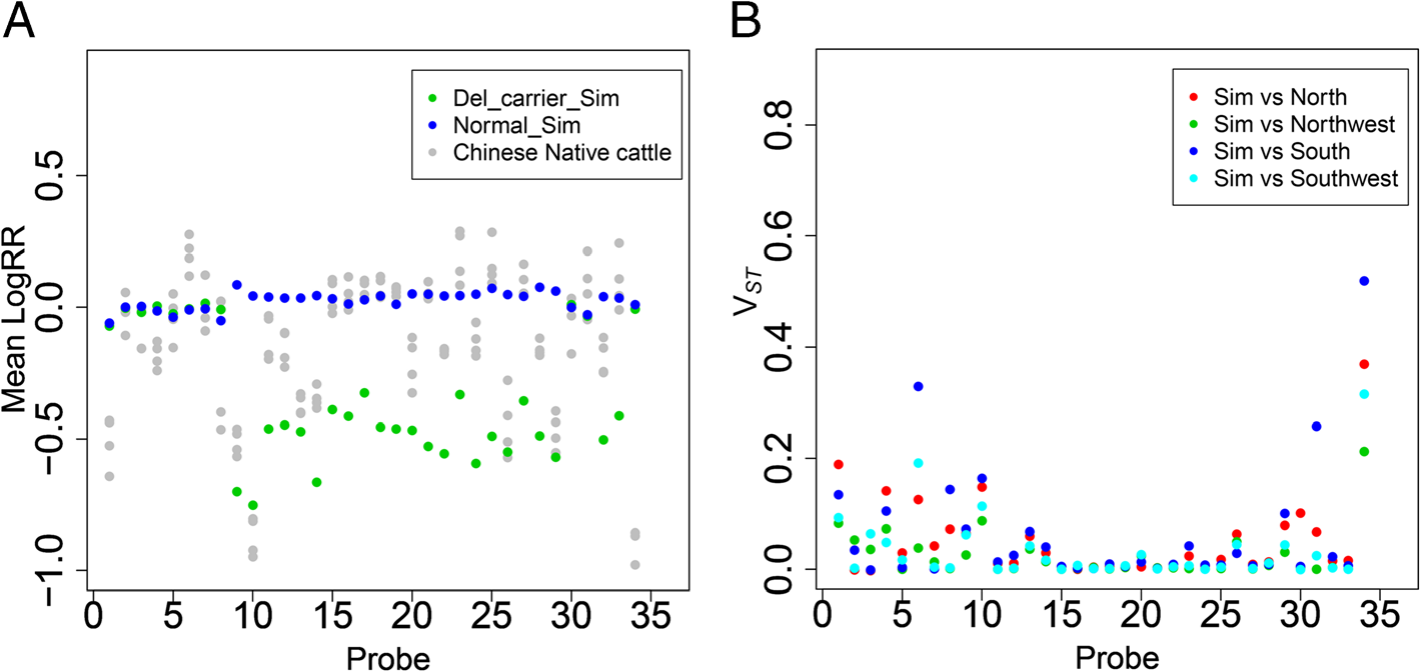
**a** Mean LogRR plot of the CNV region at BTA12. Each point shows the mean LogRR of three groups: Simmental with CNV (Del-carrier) are colored by green, Simmental without CNV (Normal) are colored by blue, while Chinese native cattle are colored by grey. **b** Estimation of *V*_*ST*_ based on LogRR for CNV regions between pairwise-groups, red points represent Simmental vs. North group, green points represent Simmental vs. Northwest group, blue color represents Simmental vs. South group, cyan points represent Simmental vs. Southwest group

To investigate the selection involved with CNVs, we further extracted the values of LogRR for the candidate regions in 188 Chinese native cattle. Notably, we found the 89 kb deletion (with 34 SNPs) located at BTA12 showed obvious difference for average LogRR between Del-carrier (i.e. Deletion carrying) individuals and Normal individuals in Chinese Simmental cattle (Fig. 3a). Using the V_*ST*_ statistics, we also obtained several peak with high V_*ST*_ values within the regions (BTA12:22–23 Mb) (Fig. 3b) for the comparison between Chinese Simmental cattle between and four groups of Chinese native cattle (North group, Northwest group, South group and Southwest group) [34]. Our result suggested this candidate CNV region for ADG with high frequency may be under positive selection in Chinese Simmental beef cattle.

**Fig. 4:**
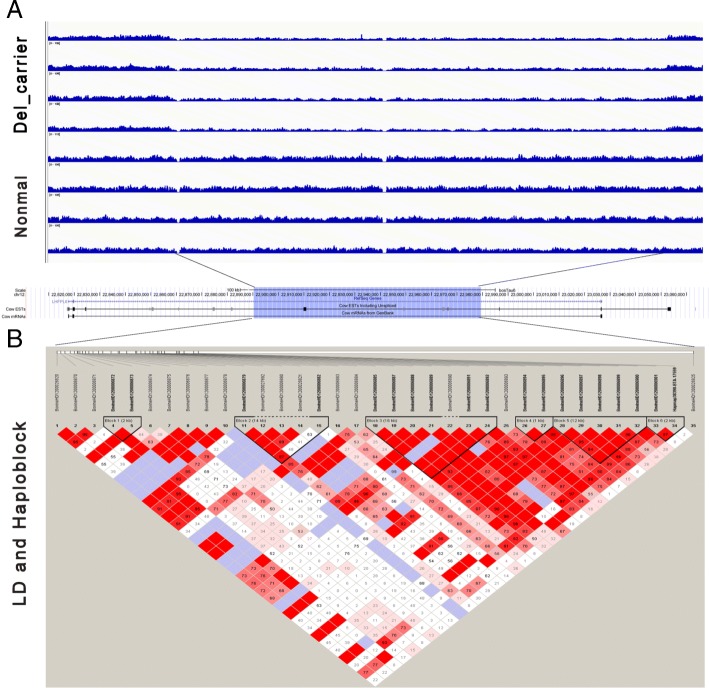
**a** Identification of an 89 kb deletion affecting ADG by genome sequencing screen captures from the IGV program depicting aligned reads around BTA12: 22890419–22,979,469 bp (top panel) after mapping to the UMD3.1 reference genome. The low read depths was indicative of deletions, which was confirmed by PCR. **b** LD plot of SNPs covering the entire CNV region at BTA12. Linkage-disequilibrium pattern across the CNV region and flanking regions for the 34 SNPs. Six haplotypes are shown as predicted by Haploview

We then extracted the whole genome sequencing reads available for four individuals with deletions as predicted by BovineHD SNP array data. Integrative Genomics Viewer (IGV, http://software.broadinstitute.org/software/igv/) was utilized to capture the changes of NGS data [35]. In all Del-carrier animals, the occurrence of deletion was obviously observed from the sequencing dataset. Notably, we found clear changes for this deletion across samples, which indicates potential copy number deletion when compared with normal samples (Fig. 4a). Next, we extracted the 34 SNPs within this region and generated the LD blocks, we observed several blocks with high LD patterns covering the right part of this deletion region (Fig. 4b). However, no candidate SNP for ADG were found from previous reports, therefore we suspected this deletion is likely be one of important structural variants that contribute to the change of ADG in beef cattle.

## Discussion

Genome wide association studies have remarkably advanced our understanding of the genetic basis of complex traits. However, these strategies cannot fully evaluate the overall heritability as other genomic variants may contribute effect for these traits [1], thus elucidation of genetic mechanism of CNV for complex traits still needs to be further investigated [36, 37].

Despite the improvements in genotyping platforms and statistic approaches have facilitated the discovery of CNVs, integrating CNVs analysis into GWAS for complex traits remains challenging. Although it is possible that CNVs are in linkage disequilibrium (LD) with associated variants, the identification of causal variants may still require us to consider CNVs beside SNPs. Previous studies of CNV association for complex trait in farm animal are mostly done using common CNVs detected by a multivariate analysis [11, 12, 38]. These approaches utilize the copy number analysis module under the multivariate option, and thus, facilitate the identification of common CNV segments. However, the CNAM algorithm force the CNV boundaries within a fixed window, which may cause CNV boundary enforcement artifacts. Compared to CNAM method, probe based association implemented in ParseCNV was developed to facilitate data processing and improve transparency for CNV association studies [27]. ParseCNV converts the individual level CNV calls into population level probe-based CNV states, thus this process can facilitate variable construction for association test based on CNV.

To systematically search for CNVs that contribute genetic architectures of ADG, we conducted a genome-wide association study based on CNVs using Illumina Bovine 770 K BeadChip in Chinese Simmental cattle. Our previous studies identified 263 CNV regions (CNVRs), which covering 35.48 Mb (1.41%) of the cattle genome in ~ 700 individuals [29]. In present study, we found 248.7 Mb, corresponding to 8.9% genome. This probably is due to larger sample size was used for CNV discovery in our populations. Large population can facilitate the application of CNV-based GWAS analysis and help to improve the detection of potentially associated CNV for ADG. In addition, PCR-based validation results showed around 86% of the validation based on qPCR were consistent with the PennCNV predictions. Also, CNV annotation indicates several genes with significant over-representation were related to receptor activity, immune and antigen processing, which are consistent with previous CNV analyses in cattle and other mammals [30, 39–42]. Totally, using probe-based CNV association analysis, we identified 38 significant probes and 7 corresponding CNV regions associated with ADG. This finding, for the first time, reported the associated CNVs contributing to ADG in farm animals. Our previous study has identified 40 significant SNPs and 7 prominent genes for ADG using multi-strategy GWAS in Chinese Simmental beef cattle [25]. Additionally, no SNPs, genes and regions in this SNP-based GWAS was found overlapped with the identified CNVs in the current study. Thus, the CNV deletions discovered in present study might contribute to ADG alone.

Totally, we have identified several candidate genes (e.g. *LHFPL6*, *SORCS3, GRB10*, *CENPW, ESR1* and *ATG14*) within or near candidate CNVs for ADG. Among them, we found one common deletion imbedded in *LHFPL6* at 22.9 Mb on BTA12 with high frequency in Chinese Simmental population. This gene belongs to a member of the lipoma HMGIC fusion partner (*LHFP*) gene family, which was reported that fused to a high-mobility group gene in a translocation-associated lipoma. Mutations in LHFP-like gene was found that related to the deafness in mice and humans [43, 44]. Moreover, we suspected the high frequency deletion occurred under positive selection and may play an important role to affect complex traits. Also, our *V*_*ST*_ statistic results suggested this deletion display significant association with ADG in Chinese Simmental cattle compared to native cattle. Therefore, this CNV may potentially act as important genome variant under selection contributing to ADG.

In addition, we identified one rare deletion near *GRB10* located at 5.1 Mb on BTA4 using both probe-based and region based association analyses. *GRB10*, growth factor receptor-bound protein 10 gene, is an intracellular adaptor protein that acts as a negative regulator of insulin and insulin-like growth factor receptors to restrict fetal and placental growth during mammalian development [45, 46]. This gene have been identified as candidate imprinted gene associated with growth-related trait in Irish Holstein-Friesian cattle [47, 48]. *GRB10* has also been reported to be related to the development of fiber number in skeletal muscle [49] and milk tridecylic acid [50]. However, the functional study of these identified deletions still need more efforts to be further explored with third generation sequencing and other experimental validations. Our analyses provided some valuable insights into the understanding the missing heritability of ADG. To our knowledge, the present study provides the first case of association between CNVs and quantitative trait in Chinese Simental beef cattle. These results extend our understanding of CNV in complex trait and pinpoint to the importance of utilizing new methods that allow for considering these variations in genome-wide association [51]. Further functional study and expression assays can be utilized to assess the biological effects of CNVs in candidate genes and help to understand their contribution to complex traits in farm animals.

## Conclusions

Our study identified 24 and 12 significant SNP probes within four deletions and three duplications for ADG, respectively. Among them, we found one common CNV deletion with 89 kb imbedded in *LHFPL6* at 22.9 Mb on BTA12, this deletion was not overlapped with any candidate SNP for ADG compared with previous SNPs-based association studies, suggesting its important role for ADG. In addition, we identified one rare deletion near *GRB10* at 5.1 Mb on BTA4 for ADG using both probe-based association and region-based approaches. Our results provided some valuable insights to elucidate the genetic basis of ADG in beef cattle, these findings offer an alternative perspective to understand the genetic mechanism of complex traits in terms of copy number variations in farm animals.

## Methods

### Ethics statement

No ethics statement was required for the collection of genetic material. The data from animals included in this study were derived from previous analyses that obtained specific permissions [25].

### Samples and phenotype data

Samples were genotyped using Illumina Bovine HD SNPs array. A more detailed description of the original array data set can be found in our previous publication [25]. The resource population consisted of 1173 Simmental cattle that were born between 2008 and 2013 in Ulgai, Inner Mongolia. After weaning, all calves were transferred to a fattening farm in Beijing and fattened in the same pens for 8~12 months. All animals were fed with same feeding and management conditions, and ADG was estimated during the fattening period. Test distribution of ADG trait showed it follow a normal distribution and analysis of variance (ANOVA) showed that farm, sex, year of measurement, fattening days had significant effects (*P* < 0.01). Thus, these factors were adjusted in the linear regression model, and the resulting trait residual was further considered for ADG association test.

### CNVs detection

PennCNV v1.0.4 software was utilized to identify CNV across autosomes [26]. PennCNV incorporates both the Log R Ratio (LogRR) value and the frequency of allele B (BAF) for CNV detection. The CNV calling was carried out following the previous study by Yang et al. [34]. The final CNV events were produced by keeping high quality samples according to the following criteria: call rate > 0.95, standard deviation (SD) of LRR < 0.35, and GC waviness factors as 0.005.

### CNV association analysis

To identify CNV regions associated with ADG, CNV calls and quality measures were translated to probe level using ParseCNV [27]. ParseCNV proposes an integrative CNV association method that convert CNV calls into probe-based statistics for individual CNVs. As CNV boundaries vary across individuals, the beginning and end points of CNVs may be unclear, we are not able to classify different CNVs as identical or different, thus CNV association test were performed at the probe level.

We tested the frequency of SNP probes affected by various CNV types separately, i.e. deletions, duplications and genomic regions affected by both types of CNV. The association between CNV carrier frequencies and ADG across population were evaluated using linear mixed model implemented in EMMAX software [52]. Relatedness among individuals was utilized as random effects based on SNPs genotype. For CNV association, a suggestive genome wide threshold was considered in present study as suggested by [27]. The probe-based statistical significance (−log_10_ *P*-value) of neighboring probes were calculated using EMMAX method. Then the neighboring SNPs with comparable significance were collapsed into CNVRs which constitute genomic span of consecutive probes (at less two probes). The local lowest *P*-value for identified probes was used to represent the significant level of association of CNVR. Accordingly, a multiple correction was carried out for each probe using *qvalue* package [53], and *q* value < 0.05 was used to determine level of significance.

### Region-based CNV association analyses

We next utilized the density of probes within CNV regions to assess the possible enrichment of region-based CNVs. The cumulative burden of CNVs can be effectively estimated on a region level using the approach implemented in CNVtools [28]. It combines the information across CNV probes to obtain a one-dimensional signal using principal component and Bayesian information criterion for each sample. A copy number genotype was assigned to each locus for each individual to test for genetic association with a quantitative trait based on a standard regression approach. The exact boundaries of the candidate regions were based on the BosTau6 (UMD 3.1) reference assembly.

### Pathway analysis and CNV genes annotation

We searched the genes affected by the identified CNVs using UCSC genome browser (UMD 3.1). Any refSeq genes that was either fully included or broken by CNV that were considered as CNV affected. To evaluate the effects of disrupted genes from any particular functionally defined molecular pathway, we investigated the CNV-disrupting genes using the DAVID gene functional classification system [54]. Deletion and duplication were considered separately. To avoid false positives, we further considered that enriched pathway which have at least two genes and the *P* value < 0.05 after the Bonferroni correction for multiple testing.

### CNVs overlapped with QTLs associated with ADG traits

QTLs information were downloaded from cattle QTLdb [55]. We merged all QTL regions into a set of unique non-redundant regions. The coordinates of QTLs based on Btau_4.0 were converted to UMD3.1. The liftOver conversion between assemblies was conducted at a relaxed threshold (Minimum ratio of bases that must remapped was set to 75%).

### Next generation sequencing analysis

Genomic DNA from four Chinese Simmental bulls was extracted from blood samples using a TIANamp Blood DNA Kit (Tiangen Biotech Company limited, Beijing, China), and DNA with an A260/280 ratio between 1.8 and 2.0 were subjected to further library construction. Two paired-end libraries were constructed for each individual, the read length was 2 × 150 bp, and whole genome sequencing was performed using Illumina Hiseq2500 instruments (Illumina Inc., San Diego, CA, USA). All processes were performed according to the standard manufacturer’s protocols. Each sample was sequenced to an approximate coverage of 20X. We removed low-quality reads following filters: (1) reads with an adaptor, (2) reads containing more than 10% unknown bases, (3) reads containing more than 50% low-quality bases. After filtering, we used the bwa-0.7.8 with parameters (mem -t 4 -k 32 -M) to perform sequence alignment based on the UMD3.1 genome assembly [56].

### Quantitative PCR validation

Quantitative PCR (qPCR) was utilized to validate seven associated CNVs detected by PennCNV. For each CNV, primers were designed using Primer3 web tool (http://bioinfo.ut.ee/primer3-0.4.0/primer3/). To ensure the amplification efficiencies, standard curve of each pair of primer was generated using template from serial diluted genomic DNA sample of a common cattle. The Basic Transcription Factor 3 (*BTF3*) gene was selected as the control assuming two copies of DNA segment. With a total volume of 20 μL reagents in a 96-well plate, qPCR was conducted using SYBR green chemistry in triplicate reactions on ABI STEPONE plus, thermo Real-Time PCR System. The condition for thermal cycle was as follows: 2 min at 95 °C followed by 40 cycles at 95 °C for 10 s, 60 °C for 40 s. We calculated the relative copy number for each selected region using the 2^-ΔΔCT^ method. First, the average CT value of three replications of each sample and normalized against the control gene, then ΔCT value was estimated between the CNV carrier sample and a reference sample with normal status.

## Additional files

Additional file 1: Summary of identified CNV and CNVRs using PennCNV in Chinese Simmental beef cattle. (XLSX 3459 kb)

Additional file 2: Gene annotation of duplications and deletions for overlapped genes, CDSs and exons. (XLSX 309 kb)

Additional file 3: Gene ontology (GO) enrichment using DAVID for CNVs. (XLSX 233 kb)

Additional file 4: Deletion and duplication regions overlapped with the merged QTL regions for ADG. (XLSX 37 kb)

Additional file 5: Summary of probe-based CNV association analysis results including probe name, chromosome, position, *P*-value, and adjusted q values. (XLSX 3735 kb)

Additional file 6: Primers information and qPCR validations of seven CNVs. (XLSX 11 kb)

## Abbreviations

ADG: Average daily gain
CNVR: Copy number variation region
BAF: Frequency of allele B
BTA: *Bos Taurus* autosomes
CNV: Copy number variation
GWAS: Genome-wide association study
IVG: Integrative genomics viewer
LogRR: Log R ratio
QTL: Quantitative trait locus
SNP: Single nucleotide polymorphism
